# Population Dynamics of Autocatalytic Sets in a Compartmentalized Spatial World

**DOI:** 10.3390/life8030033

**Published:** 2018-08-18

**Authors:** Wim Hordijk, Jonathan Naylor, Natalio Krasnogor, Harold Fellermann

**Affiliations:** 1 Institute for Advanced Study, University of Amsterdam, 1012 WX Amsterdam, The Netherlands; 2 Interdisciplinary Computing and Complex BioSystems Research Group (ICOS), School of Computing, Newcastle University, Newcastle upon Tyne NE98, UK; j.r.d.naylor@newcastle.ac.uk (J.N.); natalio.krasnogor@ncl.ac.uk (N.K.); harold.fellermann@newcastle.ac.uk (H.F.)

**Keywords:** protocells, origin of life, autocatalytic sets, evolvability

## Abstract

Autocatalytic sets are self-sustaining and collectively catalytic chemical reaction networks which are believed to have played an important role in the origin of life. Simulation studies have shown that autocatalytic sets are, in principle, evolvable if multiple autocatalytic subsets can exist in different combinations within compartments, i.e., so-called *protocells*. However, these previous studies have so far not explicitly modeled the emergence and dynamics of autocatalytic sets in *populations* of compartments in a spatial environment. Here, we use a recently developed software tool to simulate exactly this scenario, as an important first step towards more realistic simulations and experiments on autocatalytic sets in protocells.

## 1. Introduction

Autocatalysis, i.e., the ability of molecules to catalyse their own synthesis, is a hallmark of virtually any origin of life scenario, since it is the chemical equivalent to biological replication—the fundamental feature of living entities to “make more of themselves”. However, such autocatalytic molecules, directly catalysing their own production, are rare, and it is unlikely that life kick-started with such “selfish” autocatalytic chemicals. However, autocatalysis can also be obtained at a systems level, if a chemistry features a set of mutually catalytic molecules in which the formation of every member is catalysed by other members of the set. Such a set is then able to collectively catalyse all its constituents, even if none of its members is a true (i.e., direct, or “selfish”) autocatalyst [1,2,3].

The study of such collectively autocatalytic sets (CAS) has revealed that they are likely to emerge spontaneously in sufficiently diverse chemistries, even under modest catalytic activity [4], and that they are able to dynamically upconcentrate their members in order to maintain themselves. While CAS are able to draw resources from potential competitors [5], they have been criticised for displaying little to no evolvability [6]. The argument goes that once an autocatalytic cycle establishes in a random chemistry, there is nothing that destabilises this cycle in order to make room for the emergence of other autocatalytic cycles, since the concentration of all involved molecules increases exponentially. Even if a random chemistry allows for multiple catalytic cycles as hypothetical individual units of selection, these would eventually just coexist, leaving no room for further evolutionary adaptation.

It has been suggested that the limited evolvability of CAS could be overcome by embedding autocatalytic sets into compartments [2,7]. Such encapsulated reaction systems are able to draw resources from and potentially release products back into the environment. Encapsulation with environmental coupling, according to the claim, might reconstitute selection among competing CAS, since different compartments can host different active autocatalytic cycles, which can be destabilised through resource competition and random fluctuations during compartment division. As Kauffman, who introduced the concept of CAS, writes [2]: “Theoretical work and experimental work on CAS both support their plausibility as models of openly evolvable protocells, if housed in dividing compartments such as dividing liposomes”. This intuition has recently been confirmed by computational investigations that put CAS into flow reactors in order to mimic encapsulation in semi-permeable compartments [7].

Encapsulated reaction systems have been studied extensively in the origins of life context under the term protocells [8,9,10]. Protocells are simple metabolisms occurring within compartments (e.g., lipid or fatty acid vesicles) that have the capacity for growth and self-replication. Potentially equipped with inheritable chemical “information”, they are generally regarded as primitive units of (limited) evolution (through compositional inheritence), eventually leading to true open-ended Darwinian evolution. While protocells have not yet been fully implemented in the laboratory, both theoretical and experimental investigations have uncovered numerous necessary requirements about the involved chemicals and their coupling [11,12,13,14,15,16].

Related to protocells are models from the realm of the lipid world, where an explicit covalent metabolism is replaced by conceptually simpler cross-catalytic association and dissociation dynamics of compartment forming amphiphilic molecules [17]. It has been demonstrated that, even in such lipid models, random network properties of the constituting molecules can give rise to heredity [17], speciation [18], and population dynamics [19], although it has been argued whether systems lacking covalent chemistry are able to undergo full evolution [7,20].

So far, though, relatively little attention has focused on studying collectively autocatalytic sets as metabolisms and inheritable information for protocells. A few studies have shown that such “autocatalytic protocells” indeed have the ability, in principle, to synchronise their internal metabolism and membrane dynamics, and may evolve [5,7,21,22]. However, these studies on autocatalytic sets did not explicitly model populations of protocells in a spatial environment. Here, we make an important first step in this direction by using a recently developed software tool to simulate the emergence and dynamical behavior of autocatalytic sets in a population of simple compartments that exist in a spatially explicit world. We present several illustrative initial results, discuss how these could be relevant in the context of the origin and early evolution of life, and provide suggestions for further work, in combination with experimental studies.

## 2. Background

The concept of autocatalytic sets was originally introduced by Kauffman [23,24,25], and subsequently formalised and further developed as RAF theory [4]. An autocatalytic set (or RAF set) is defined as a set R of reactions and associated molecules that is:

1. *Reflexively autocatalytic* (RA): each reaction in R is catalysed by at least one molecule from R itself; and
2. *F-generated* (F): all reactants in R can be created from some food set *F* by using a sequence of reactions from R itself.

The food set *F* is a set of molecule types that are assumed to be available from the environment. This notion of autocatalytic sets has been defined mathematically more rigorously, and an efficient (polynomial-time) algorithm for finding RAF sets in general reaction networks has been developed [26,27,28]. RAF theory has been applied extensively to simple polymer-like models of chemical reaction networks, showing that autocatalytic sets are highly likely to exist at chemically realistic levels of catalysis, and under a wide variety of model assumptions [5,26,27,29,30]. Importantly, these results show that autocatalytic sets often consist of a hierarchy of smaller and smaller autocatalytic subsets, i.e., smaller subsets of reactions that themselves are RAF sets [28,31]. Finally, the formal RAF framework has also been applied successfully to analyse real chemical and biological reaction networks [32,33].

**Figure 1:**
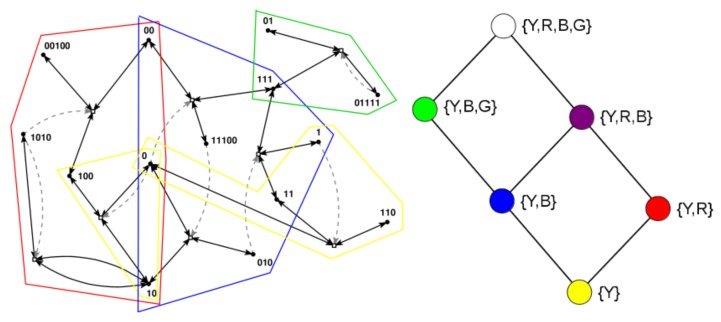
RAF set example. **Left**: an example of an RAF set as found in an instance of the binary polymer model. Black dots (labeled with bit strings) represent the molecule types, and white boxes represent reactions. Solid black arrows indicate molecules going into and coming out of a reaction, while dashed gray arrows indicate catalysis. Coloured polygones indicate some of the RAF subsets (see text). **Right**: the six closed RAFs (colour coded) and their mutual subset relationships.

Many of these earlier results are based on a simple model of reaction networks known as the binary polymer model [34]. In this model, molecules are represented by bit strings up to a maximum length *n*, with the food set consisting of all bit strings up to a small length *t* (usually t=2, i.e., the monomers and dimers). The chemical reactions consist of the possible ligations (gluing two bit string together into a longer one) and cleavages (cutting a bit string into two smaller ones). Finally, catalysis is assigned randomly, with a fixed probability *p* that a given molecule (bit string) catalyses a given reaction (a ligation and its corresponding cleavage). Figure 1 (left) shows an example of an RAF set R, consisting of eight reactions, that was found in an instance of the binary polymer model with n=5, t=2, and p=0.0045, with some of its RAF subsets indicated by the coloured polygones.

Note that catalysis is considered an “all-or-nothing” feature in this context. However, (relative) catlaysis rates can also be taken into account [35], as well as inhibition, i.e., molecules that *prevent* reactions from happening [28,36]. In the simulations described below, (relative) reaction rates are explicitly used, and in one instance a form of inhibition is also included.

Recently it was argued that the main RAF subsets of interest, in particular in the context of the origin of life, are the so-called closed RAF sets, in which all reactions for which a catalyst is present (given the set of molecule types currently present in the system) are included [30,37]. For example, when only food molecules (monomers and dimers) are present initially, only the reactions within the yellow RAF subset can proceed catalysed. This yellow subset thus forms the smallest closed RAF (the “yellow” closed RAF), and is (necessarily) always part of any larger closed RAF as well.

However, for the three reactions in the red RAF subset, not all catalysts are present yet when only the yellow subset exists. One of its reactions will have to happen uncatalysed to create the required but missing catalyst spontaneously. Of course, all reactions can happen without a catalyst, but they do so at a lower rate, which means there is usually some (stochastic) waiting time before this happens. Once it does happen, though, the red subset comes into existence, with all its reactions proceeding catalysed. Thus, the yellow and red subsets combined form another closed RAF (the “red” closed RAF).

Similarly, the blue subset requires any one of its three reactions to happen spontaneously before the full subset can come into existence. When it does, the yellow and blue subsets combine to form yet another closed RAF (the “blue” closed RAF). When the yellow, blue, and red subsets all exist, they form an even larger closed RAF (the “purple” closed RAF).

Finally, the green subset, which also requires a spontaneous (uncatalysed) reaction reaction, can form an extension of the blue RAF subset, but only once the blue subset itself already exists. Thus, the yellow, blue, and green subsets combined form a closed RAF as well (the “green” closed RAF). When all subsets (yellow, red, blue, and green, i.e., the full autocatalytic set R) exist, they form the largest possible closed RAF (the “white” closed RAF).

These six possible closed RAF sets are shown in the diagram in Figure 1 (right) with their respective colours, the combination of RAF subsets they are made up of, and where an edge between two nodes means that the closed RAF at the lower end of the edge is a direct subset of the closed RAF at the upper end of the edge.

Earlier, it was shown that the autocatalytic set R in Figure 1 (left) actually contains 29 RAF subsets, but that only six of these are closed RAFs (the ones indicated here) [37]. Therefore, from a dynamical point of view, the other 23 RAF subsets are of little interest, as they would immediately expand into the larger closed RAF that they are part of. In other words, RAF subsets that are not closed are transient, whereas closed RAFs are stable over long time spans, until some spontaneous but rare reaction happens that allows an even larger closed RAF to come into existence.

The RAF set R as shown in Figure 1 (left) and its six closed RAFs as shown in Figure 1 (right) are used here to illustrate, through computer simulations, the emergence of different autocatalytic (sub)sets in a population of compartments that exist in an explicit spatial environment.

## 3. Methods

Simulations were performed with the Simbiotics package [38]. Simbiotics is a multicellular simulator which represents cells as individual physical entities embedded in chemical gradients, where each cell can have defined dynamics and can interact with its environment as well as other cells. The simulation toolkit provides a versatile set of data collection and analysis tools, and can be easily extended with user-defined modules and libraries. A more detailed description of Simbiotics is available elsewhere [38]. Here, we briefly describe the particular features used in this study.

We model the simulation domain as a 2D rectangle with periodic boundary conditions. Chemicals in the environment (e.g., food molecules) are defined as continuous fields over this rectangle and the usual Fick law is taken to express their diffusion. For computational purposes, Simbiotics rasterises this space into a grid of finite sized voxels and integrates the deterministic diffusion and decay dynamics using a finite difference method. A voxel is a sub-area of the 2D simulation domain, which stores the chemical quantities which exist there, enabling for the representation of localised concentrations and chemical fluxes between neighbouring voxels.

A constant flow of monomers and dimers, which we regard as food molecules, is provided by introducing these molecules at the center of the grid at a given constant rate, which then diffuse to neighboring grid locations depending on a given diffusion rate. Molecules diffuse from higher concentrations to lower concentrations. Food molecules in the environment decay with a given constant rate which models outflow of the environment. Depending on the inflow, diffusion, and decay rates, in the absence of any other dynamics, a steady state in the concentration of food molecules over the entire grid is eventually reached.

We introduce compartments by randomly placing spheres of constant radius into the space. Compartments are not allowed to overlap and are immotile throughout the simulation. Each compartment can hold molecules in their interior. Molecules are allowed to permeate compartment membranes if their lengths do not exceed a certain threshold. Permeation is proportional to the concentration difference between the compartment interior and the surrounding local environment (taken as the concentration at the grid cell that the compartment resides in), the compartment surface area, and a permeation rate constant. The actual number of molecules permeating the membrane is sampled from a Poisson distribution whose mean is the permeation rate. In the following, we allow bit strings of up to length two (i.e., all food molecules) to permeate membranes, whereas longer strings are strictly contained within compartments.

**Figure 2:**
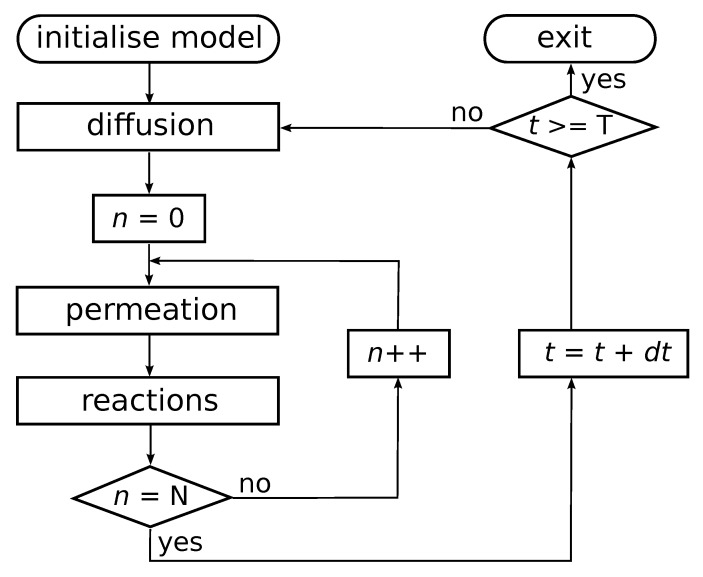
Flow diagram of the simulation algorithm. After initializing the model with N compartments, the outer loop iterates the system through time. In each iteration, the algorithm first solves for diffusion and decay of molecules in the environment using a finite difference approximation to the Fick equation. Then, for each compartment in the simulation, intracompartment molecular counts are updated by first solving permeation processes, and then running a Gillespie algorithm within each compartment. The algorithm is stopped after T time units have been simulated.

Chemical reactions within compartments are based on the reaction network presented in Figure 1 (left), i.e., an autocatalytic set that occurs in an instance of the binary polymer model. For simplicity, the simulations only consider ligation reactions. For each such reaction, an uncatalysed and a catalysed instance is included, but with different rate constants such that the rate constant for the uncatalysed reaction instance is lower than that of the catalysed reaction instance. The actual chemical dynamics within each compartment is simulated using Gillespie’s stochastic simulation algorithm [39,40]. A flow diagram of the simulation algorithm is presented in Figure 2.

Note that we do not simulate chemical reactions in the environment. This can be justified by assuming that, even if some reactions would take place in the environment, the reaction products would mostly diffuse away and out of the environment, and no real sustained chemistry would be possible, other than inside compartments [21].

**Figure 3:**
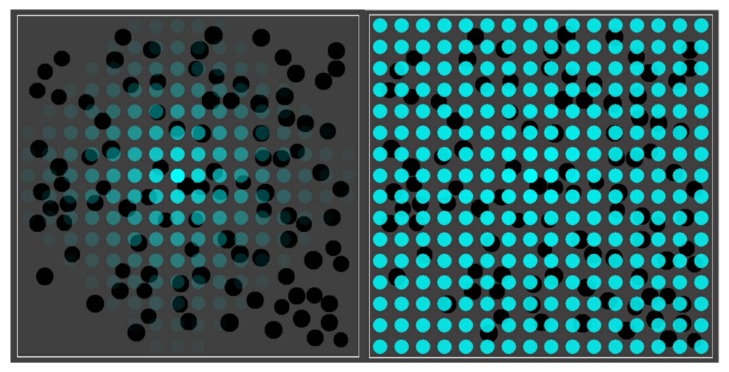
The basic simulation setup. A 16 × 16 grid with 100 randomly distributed compartments (black spheres) and concentration of food molecules (blue spheres) throughout the grid. **Left**: shortly after starting the simulation. **Right**: after an equilibrium distribution of food molecules has been reached.

Figure 3 shows the basic simulation setup at two different time steps. The spatial environment consists of a 16 × 16 grid, with 100 randomly distributed compartments (black spheres). Blue spheres indicate the (relative) concentration of food molecules in each of the grid locations. Faint (transparent) spheres indicate low concentration, and bright (solid) spheres indicate high concentration (relative to the grid location with the highest current concentration). Early on during the simulation (left frame), the food molecules are just starting to diffuse throughout the grid, while being introduced into the environment at a constant rate in the center of the grid. Later on during the simulation (right frame), an equilibrium distribution of food molecules as been reached. Food molecules will also have entered the compartments, but there is no actual chemistry going on yet (i.e., all reaction rate constants have been set to zero).

In most of the simulations presented below, the rates of food inflow, diffusion, and decay were set such that, once the equilibrium phase has been reached, there are about five molecules of each food type (i.e., monomers and dimers) within each compartment when no chemistry takes place. When the reaction rate constants are set to positive values, though, reactions will happen within compartments (starting from the food molecules). Over time, the different closed RAFs will appear inside compartments, and each compartment is coloured according to which closed RAF it currently contains (with a molecule count threshold of two), using a colour scheme as in Figure 1 (right). Detailed parameter settings for each of the simulations are given in Appendix A.

It should be noted here that, since the simulated chemical reaction networks come from an abstract polymer model, time and volume are arbitrary. In other words, the time units could, in principle, represent seconds, hours, or even days. However, what is important here is the relative difference between rate constants of catalysed and uncatalysed reactions. The absolute magnitude of these rate constants were chosen such that a single simulation takes just a few minutes, rather than hours. However, the parameters can easily be scaled up or down to change the absolute time scale. The overall behavior would remain the same, though.

Similarly, molecular quantities are somewhat arbitrary. We have chosen a threshold of two product molecules to exist before an autocatalytic subset is considered to be present. The idea behind this is that the first product generally has to be produced through a spontaneous reaction, but if there are two or more products, it is highly likely that they have been produced through catalysed reactions, and that the corresponding autocatalytic subset indeed is present in full. Such low molecular counts may seem unrealistic, but again, since the units in the system are arbitrary, these could also be interpreted as, e.g., micromolar quantities. Moreover, the origin of life most likely did happen in a low molecular-count scenario.

Simbiotics is implemented in Java and runs on all operating-systems which support the Java Virtual Machine (JVM). The software package is available for download at http://ico2s.org/software/simbiotics.html and includes a user manual that explains how to install and run it. Specific input files, specifying all relevant parameter values (including reaction rate constants), for each of the simulations described below can be downloaded from http://ico2s.org/data/extras/compartments/, which also contains the movies referred to in the results section.

## 4. Results

### 4.1. Dynamics of a Single Compartment

Before presenting results on simulating a population of compartments, we start by showing the kinds of dynamical behavior that can occur within a single compartment. This will help in understanding the subsequent results.

**Figure 4:**
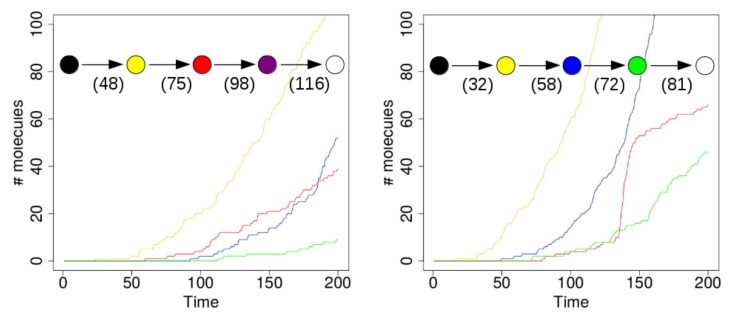
A single compartment. **Left**: a simulation run with a single compartment where the red RAF subset appears first. **Right**: a simulation run with a single compartment where the blue RAF subset appears first. Insets: the sequence of compartment colour changes in the simulations. Numbers indicate at which time steps during the simulation the respective colour changes happened.

Figure 4 shows the results of two simulations with just a single compartment, located at the center of the grid (i.e., where the food molecules flow in). On the horizontal axis in these plots is time (in arbitrary units), and the vertical axis shows total number of molecules. The yellow line represents the number of molecules of type 110 that exist inside the compartment over time. Recall from Figure 1 (left) that this molecule type (bit string) is a product of the yellow RAF subset, but one that is not used up in any other reaction. Similarly, the red line represents the number of molecules of type 00100 inside the compartment. This molecule is produced by the red RAF subset, but not used in any reactions. The blue line represents the number of molecules of type 11100, produced by the blue RAF subset. This molecule acts as a catalyst, but not as a reactant in any of the reactions, and is thus also not used up. Finally, the green line shows the number of molecules of type 01111, which are produced (but not used up) by the green RAF subset (although they are also a catalyst).

In the simulation that produced the results shown in the plot on the left of Figure 4, the first molecule of type 110 (a “yellow” molecule) is produced at time step 23. At the start of the simulation there are not many food molecules yet, as they only just start flowing into the environment. Thus, it takes a while before the first (catalysed) reactions will actually start happening, once enough food molecules are present. The second molecule of type 110 is produced at time step 48, at which point the compartment in the simulation visualization turns from black to yellow. In other words, once there are at least two molecules of type 110, the compartment turns yellow, indicating that the yellow closed RAF (consisting of the yellow RAF subset in Figure 1 (left)) is currently present inside the compartment.

However, for the other RAF subsets (red, blue, and green in Figure 1 (left)), a spontaneous reaction is required first, as explained above. Since these spontaneous (uncatalysed) reactions happen at a lower rate than the catalysed ones, there is an additional waiting time before any of these RAF subsets comes into existence. In the plot on the left in Figure 4, by chance the red subset comes into existence first, due to a spontaneous reaction. As soon as at least two molecules of type 00100 exist inside the compartment (which happens at time step 75 in this simulation), the compartment turns from yellow to red, indicating that the red closed RAF (i.e., the yellow and red RAF subsets combined) is currently present inside the compartment.

Similarly, the blue RAF subset comes into existence after one of its reactions has happened spontaneously, and as soon as at least two molecules of type 11100 are present (which happens at time step 98), the compartment turns purple, indicating that the purple closed RAF (i.e., the yellow, red, and blue subsets combined) is now present inside the compartment. Finally, the green subset comes into existence (which can only happen once the blue subset exists), and a second molecule of type 01111 is produced at time step 116, at which point the compartment turns white, indicating that the white closed RAF (i.e., all RAF subsets together) is now present. In short, the compartment has gone through the sequence of colours as shown in the inset in Figure 4 (left), where the numbers underneath the arrows indicate at which time step the change in colour happened.

However, in the second simulation (shown on the right in Figure 4), there is a different sequence of events. In this case, the blue subset comes into existence first, then the green one, and finally the red one. Thus, the compartment goes through the sequence of colours as shown in the inset of Figure 4 (right). Also note that the time steps of the changes are different between the two simulations, showing that it truly is a stochastic process.

In these simulations, the difference between the rate constants for catalysed and uncatalysed reactions is kept relatively small (about one order of magnitude), so that the changes in colour actually happen within a reasonable amount of time. However, in real chemical systems, this difference will generally be larger (often several orders of magnitude [41]), so the waiting times between colour changes (i.e., new RAF subsets coming into existence) will also be much larger. In fact, in principle, it could even be the case that only the red closed RAF actually comes into existence, but never the blue one, or vice versa, if the required spontaneous reaction never happens within the total simulation time.

These simulations confirm the postulated lack of evolvability [6] of this particular RAF: once an autocatalytic set comes into existence, it continues to catalyse its members, which are never consumed by future reactions. No matter the chain of events, the compartment will ultimately display the fully developed white RAF. If the presence or absence of RAF subsets are taken to be evolutionary traits, these traits can never be selected against in evolutionary competition dynamics. With the basic one-compartment dynamics explained in detail, we can now move on to populations of compartments.

### 4.2. Dynamics of a Population of Compartments

**Figure 5:**
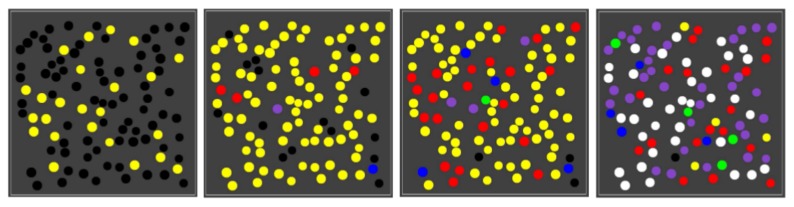
A population of compartments. Four snapshots over time from a simulation with 100 compartments.

Using the same parameter values (including the reaction rate constants), we next ran the same simulation, but with 100 compartments randomly spread out in the grid. As the one-compartment simulation already suggests, different compartments in the population go through different sequences of colour changes at different times, giving rise to a population of mixed compartment “types” (i.e., some with only the yellow closed RAF, some with the red closed RAF, some with the blue, etc.). Figure 5 shows four snapshots from one such simulation.

As the figure shows, early on most compartments are still black, i.e., no chemistry is going on yet, but some are have already gathered enough food molecules to have the yellow closed RAF in existence. A little later, most compartments are in the yellow state, and there are also already a few red, blue, or purple compartments. This compartment diversity then increases, until finally most compartments have turned purple or white, although there are also still several other colours, including a few yellow ones. A movie of this simulation (for 150 time units) is provided at the following web page: http://ico2s.org/data/extras/compartments/, where a copy of the used parameter file can also be viewed or downloaded.

Szathmáry, Kauffman, and colleagues have shown that such variability is exactly one of the main conditions for autocatalytic sets to be evolvable [7]. Having different combinations of autocatalytic subsets (i.e., different closed RAFs) existing inside different compartments can give rise to competition for resources between compartments, and new autocatalytic subsets coming into existence in some compartments, due to rare spontaneous reaction events, give rise to variation.

These mutations are immediately evident in the movie as the changes in colour of the compartments. Competition between compartments is less evident, but is also present. Note that, even at the end of the simulation, there are still a few yellow and even one black compartment. Because other compartments already have larger closed RAFs existing inside them, those other compartments are using up food molecules at a relatively high rate. Given that molecules are introduced at a constant rate and then diffuse through the grid, they tend to diffuse mostly towards those compartments that use them up at high rates, simply due to the resulting concentration differences. Therefore, food resources are diverted away from compartments that do not have much chemistry going on yet (e.g., they may only have the yellow closed RAF present), and are therefore “starved”, becoming even less likely to ever go beyond the black or yellow state. A similar type of competition was shown to exist in principle, in a one-compartment scenario with two competing RAF subsets, in earlier simulation studies [5].

Note, though, that, in the reaction network used in this simulation, only ligation reactions are included, but not cleavage reactions. Therefore, mutations can only happen in one direction: only new autocatalytic subsets can come into existence, giving rise to a larger closed RAF existing inside a compartment. As such, the lack of evolvability observed in the last section is not automatically remedied by investigating populations of compartments. What would make the dynamics more interesting is a mechanism for mutations where an autocatalytic subset is lost.

### 4.3. The Influence of a Toxic Element

**Figure 6:**
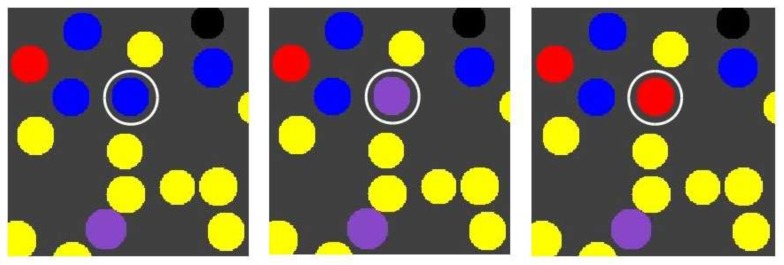
The influence of a toxic element. The production of a toxic element by the red RAF subset can cause the blue RAF subset to be lost again, making a compartment change from blue to purple to red (indicated by the white circle).

Suppose that molecule type 00100, produced by the red RAF subset, can spontaneously turn into a “toxic” element that catalyses the destruction of molecule type 11100, which is produced by and acts as a catalyst of the blue RAF subset. In other words, once the red RAF subset is present, it can suppress the existence of the blue RAF subset. This way, it is possible to have “mutations” where an autocatalytic subset is lost. Such an event is illustrated in Figure 6.

The three images in Figure 6 show snapshots of the same area in the grid, but at different time steps, from a simulation that includes the toxic element, and where the reaction making up the green RAF subset is left out (for illustrative purposes). All other parameter values are kept the same as in the previous case.

Note that the compartment indicated by the white circle changes from blue to purple to red. In the previous simulation, this would not have been possible, since concentrations of long polymers cannot decrease. However, what happened in this simulation is that the given compartment acquired the blue RAF subset first (becoming blue) and then the red one (becoming purple), but then the toxic element produced by the red subset destroyed the blue subset, causing the compartment to change to red. On the other hand, the red subset can also (temporarily) destroy itself. If all the molecules of type 00100 produced by the red subset turn into toxic elements, the red subset itself does not exist anymore either, until new 00100 molecules are produced. Thus, a compartment could also oscillate between yellow and red. Both of these situations happen in various locations and at various times in the simulation, a movie of which (for 100 time units) is available on the mentioned web page.

Another way to see the influence of the toxic element, compared to the base case from the previous subsection, is to look at the number of compartments of each type (i.e., colour) over time. Figure 9 (left) shows such a comparison for two representative simulations for each case. As this plot shows, in the toxic element case (dashed lines), the number of purple compartments is clearly suppressed, as it is now more difficult to have the red and blue RAF subsets existing simultaneously.

Taken together, the simulations presented so far demonstrate that populations of RAF sets allow for competition and selection dynamics and ultimately for (limited) evolution, if the chemistry allows for molecules of RAF sets to be consumed. We next showcase some other dynamical features that can occur in spatially embedded populations of compartmentalised RAF sets.

### 4.4. The Influence of a Permeable Inducer

So far, the various compartments do not interact with each other or the environment, other than taking up food molecules. In the next simulation, we also include the secretion of an element produced by the compartments, in particular one that can induce other compartments to acquire an autocatalytic set (if they do not already have one).

**Figure 7:**
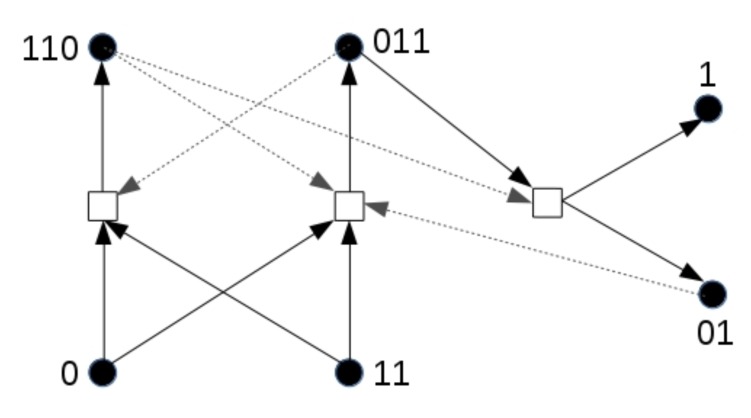
A reaction network with an inducer. The reaction network used to show the influence of an inducer (molecule type 01). As before, dashed arrows indicate catalysis.

To illustrate the effect in its simplest form, we use a different chemical reaction network, shown in Figure 7, than in the previous simulations. This network also forms an RAF set that could exist in the binary polymer model. For this simulation, only molecule types 0 and 11 are food molecules. Note that this RAF set also needs at least one spontaneous reaction to come into existence. As before, a compartment turns yellow as soon as at least two molecules of type 110 are present.

However, note that one of the products of this RAF set, molecule type 01 (not part of the food set), acts as an additional catalyst for one of the two reactions that initially need to happen spontaneously. We assume that this molecule can cross the compartment boundary since it has the same length as one of the food molecules, and then diffuse (at low rate) through the grid. What can then happen is that one compartment that already has the RAF set present secretes one or more molecules of type 01 into the environment, which then slowly diffuse through the grid and enter another compartment. If this other compartment does not have the RAF set existing yet, but it has acquired enough food molecules (0 and 11), the inducer molecule (01) can catalyse the required reaction for the RAF set to come into existence, rather than having to wait for an uncatalysed reaction.

**Figure 8:**
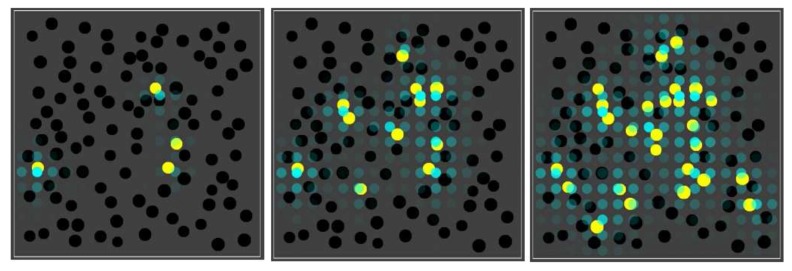
The influence of a permeable inducer. Three snapshots over time from a simulation where the RAF set produces an permeable inducer that can diffuse through the lattice. The blue spheres indicate the concentration of this inducer in the different grid locations.

In other words, molecule type 01 can act as an “inducer” for RAF sets to come into existence in nearby compartments. This situation is shown in Figure 8, where the blue spheres indicate the concentration of molecule type 01 (outside of compartments) in each grid location in the environment.

In the corresponding movie (available on the same web page again, including the parameter file), it is clear that black compartments tend to turn yellow preferentially in the vicinity of other compartments that are already yellow, where the highest concentrations of the inducer are found. The end result is that the yellow compartments are clustered, rather than distributed homogeneously throughout the space.

**Figure 9:**
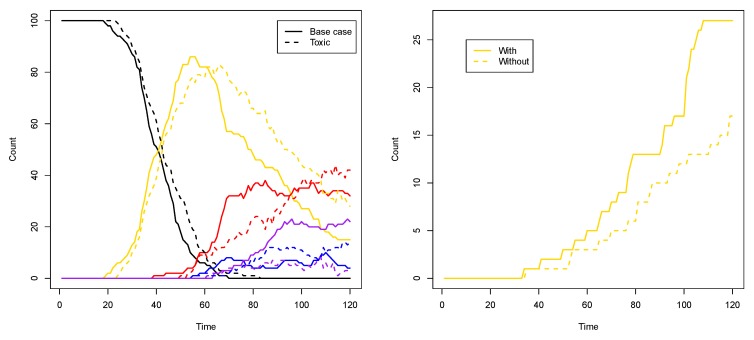
Compartment counts. **Left**: a comparison of compartment type counts between the base case and the influence of a toxic element. **Right**: a comparison of yellow compartment counts with or without the inducer.

To show that it is indeed the inducer that causes this phenomenon, we first performed ten simulations (120 time units each) where the inducer (molecule type 01) is not allowed to cross the compartment membrane. In this case, there are (on average) 16.3 yellow compartments (out of 100) at the end of the simulation. Then, we performed another ten simulations (also 120 time units each) where the inducer is allowed to cross the membrane (as in Figure 8 and the corresponding movie). In the latter case, there are (on average) 26 yellow compartments at the end of the simulation. Thus, there are significantly more yellow compartments due to the inducer (*p*-value = 0.0003). Figure 9 (right) shows a comparison of the number of yellow compartments over time between a simulation with and a simulation without the permeable inducer.

## 5. Conclusions

We have presented results of computational simulations of autocatalytic sets emerging in compartments. As far as we know, these simulations are the first demonstration of such dynamics explicitly combining (1) collectively autocatalytic sets in (2) *populations* of compartments in (3) a *spatial* environment. This provides a significant step forward towards modeling the emergence and evolution of autocatalytic sets in simple protocells.

Our simulations show that the main requirements for autocatalytic sets to be evolvable are met when encapsulating them into compartment populations: the existence of different combinations of autocatalytic subsets (i.e., closed RAFs) in a population of compartments, giving rise to different “cell types” and competition between them. Mutations in such cell types are caused by occasional new autocatalytic subsets coming into existence due to rare spontaneous reaction events, or the loss of autocatalytic subsets due to, e.g., one subset producing a toxic substance for another subset. This requirement had already been shown to be satisfied, in principle, in earlier studies [5,7], but here it is shown for the first time in a spatially explicit population setting.

We have also demonstrated that populations of encapsulated autocatalytic sets can give rise to ecological dynamics that act somewhat orthogonal to evolutionary competition and selection dynamics [42,43]. As an example of such phenomena, we have shown how permeable inducers produced in one compartment can trigger the appearance of autocatalytic sets in neighboring compartments. We regard this as an example of population dynamics, and concur that similar ecological relationships (e.g., mutualism, parasitism, etc.) should also be observable in spatially coupled populations of RAF sets.

Note, though, that the current simulations cannot yet be considered to represent true protocells. In particular, there is no coupling between the internal (autocatalytic set) dynamics and the (fixed) compartment boundary [12]. The main focus in the current study has been on the population and spatial aspects. This could, for example, model chemistry in the porous structure of hydrothermal vents [44]. However, Simbiotics can also simulate movement, growth, and division of compartments. Coupling internal dynamics with compartment growth and division is one of the hallmarks of protocell research [10,11,12,13], and will be a focus of future work.

Importantly, once implemented, compartment division and the generation of offspring will allow us to study inheritance (another requirement for evolvability [5,7]). When a compartment divides, it will distribute its biomass among its two offspring cells. Assuming sufficiently high molecular counts, both offspring cells are likely to contain all the necessary catalysts to continue the chemical dynamics of autocatalytic subsets that were already present in the parent, without having to wait for any spontaneous reactions again. However, especially at low concentrations, it might happen that one or more essential catalysts are missing in one of the offspring cells, due to stochastic fluctuations at division. In that case, one or more of the autocatalytic subsets that were present in the parent cell can be lost, which would provide another way for mutations to happen, as was already suggested [7].

Finally, although the results presented here are computational simulations using an abstract chemical reaction network, there are direct links to experimental systems. For example, recently the emergence and dynamics of autocatalytic sets of RNA molecules have been studied in microfluidics [45], which provides an experimental simulation of compartments. These RNA autocatalytic sets were created in the lab [46], and have been studied in more detail using the formal RAF framework [32]. Thus, there is a direct and natural connection between our simulations and these microdroplet experiments, which we hope to explore in future work.

Autocatalytic sets have been shown to have a high probability of existence, also for moderate and chemically plausible levels of catalysis [26,29]. Furthermore, several experimental autocatalytic sets have been constructed in the lab, either with nucleotide sequences [46,47,48] or with peptides [49]. Finally, they have been shown, in principle, to be evolvable [5,7]. Here, we have taken a first step towards a more realistic demonstration of this by simulating the emergence and dynamics of autocatalytic (sub)sets in populations of compartments in a spatially explicit environment.

Clearly, this has consequences for how we might think about the origin of life. If autocatalytic sets have a high chance of emerging spontaneously in simple compartments (e.g., lipid membranes), and can grow and evolve to become more complex, this could provide a plausible way for life to have arisen. Further simulation studies along these lines, also in combination with experimental studies as indicated, seem to be a promising avenue to shine light on this pathway to life.

## Appendix A. Simulation Parameters

The following table lists the simulation parameters used to obtained the presented results. A reaction rate c${}_{X}$ corresponds to the catalysed or uncatalysed (spontaneous) reaction with product X, and c[Y] the rate for reaction Y, as shown in the corresponding reaction graphs (Figure 1 and Figure 7).

**Table A1:** Simulation parameters.

Parameter	Value
**Figure 4, Figure 5 and Figure 6**	
Spontaneous rates	
c${}_{100}$,c${}_{110}$	0.02
c${}_{11100}$,c${}_{111}$,c${}_{010}$	0.003
c${}_{1010}$,c${}_{0111}$	0.005
c${}_{00100}$	0.001
Catalysed rates	
c${}_{11100}$,c${}_{111}$,c${}_{010}$	0.005
c${}_{1010}$	0.03
c${}_{00100}$	0.02
c${}_{0111}$	0.01
**Figure 6**	
Catalysed rates	
c${}_{01111}$	0.005
**Figure 7**	
Spontaneous rates	
c${}_{110}$,c${}_{011}$	0.0001
Catalysed rates	
c${}_{110}$,c${}_{011}$	0.05
c${}_{\left[011+110\;->\;01+1+110\right]}$	0.05
c${}_{\left[0+11+01\;->\;011+01\right]}$	0.5
**Figure 5, Figure 6, Figure 7, Figure 8 and Figure 9**	
World size	20 × 20
Compartments	100
Compartment radius	0.5
Voxel size	2.5 × 2.5
Numerical time step	0.01
**Figure 5**	
Diffusion coefficients	
D${}_{0}$,D${}_{1}$,D${}_{00}$,D${}_{01}$,D${}_{10}$,D${}_{11}$	20
D${}_{010}$,D${}_{100}$,D${}_{110}$,D${}_{111}$,D${}_{1010}$,D${}_{00100}$,D${}_{01111}$,D${}_{11100}$	10
Decay rate	
K${}_{0}$,K${}_{1}$,K${}_{00}$,K${}_{01}$,K${}_{10}$,K${}_{11}$	0.013
K${}_{010}$,K${}_{100}$,K${}_{110}$,K${}_{111}$,K${}_{1010}$,K${}_{00100}$,K${}_{01111}$,K${}_{11100}$	0
